# Navigating Remote Blood Pressure Monitoring—The Devil Is in the Details

**DOI:** 10.1001/jamanetworkopen.2024.13739

**Published:** 2024-06-03

**Authors:** Antoinette M. Schoenthaler, Safiya Richardson, Devin Mann

**Affiliations:** NYU Grossman School of Medicine, New York City, New York; Institute for Excellence in Health Equity, NYU Langone Health, New York, New York; NYU Grossman School of Medicine, New York City, New York; Institute for Excellence in Health Equity, NYU Langone Health, New York, New York; Medical Center Information Technology Department of Health Informatics, NYU Langone Health, New York, New York; NYU Grossman School of Medicine, New York City, New York; Medical Center Information Technology Department of Health Informatics, NYU Langone Health, New York, New York

The 2023 joint American Heart Association and American Medical Association statement highlighted the proven impact of remote patient monitoring (RPM) on blood pressure (BP) control and underscored the need for widespread implementation of RPM in practice-based settings.^1^ The article by Mehta et al^2^ sought to fill this implementation gap by conducting a 3-arm randomized clinical trial comparing the effectiveness of RPM of BP and medication adherence reminders, RPM of BP and medication adherence reminders with feedback to a social support partner, and usual care on reduction of systolic BP at 4 months (primary outcome). The trial included 246 adults with uncontrolled hypertension at baseline. Participants in both the RPM alone and RPM plus support person groups received a free home BP monitor, 3 text message reminders per week to take and submit their BP readings, and 1 text message to assess their medication adherence over the past week. Patients in both arms also received weekly feedback on their BP and adherence data. Support partners who opted in to the study also received weekly feedback, which was used to send the partnered patient a weekly motivational message tailored to their BP and adherence data. The intervention arms also included a summary of the BP values and recommendations for medication adjustments sent to the primary care physician (PCP) via the electronic health record (EHR) to prompt medication titrations if 3 of the 10 BP readings were elevated based on Eighth Joint National Committee guidelines. For the cohort enrolled in 2018 (151 participants), the PCP-facing intervention components were sent to the PCP directly. For the second cohort in 2019 (100 participants), the PCP-facing intervention was redesigned to send the EHR messages to a centralized nurse who then routed medication changes to the PCPs. Patients randomized to the control arm received standard care. Overall, the investigators found no significant differences between the intervention and control groups for any of the BP outcomes. These negative findings need to be evaluated within the context in which the intervention was delivered and the choices made for the intervention content.

Before discussing the limitations, it is important to point out some of the study’s strengths. First, it was conducted at an urban primary care clinic and included mainly female and Black participants. Second, the provision of a free BP monitor, the use of text messages as a ubiquitous digital platform with high reach, and the inclusion of a support partner all help to address the digital determinants of health that limit health disparity populations’ engagement with digital health interventions.^3^ Finally, the intervention was well-received and highly recommended to others who may need it. Outside of these strengths, the study had several major shortcomings related to the implementation context and intervention choices that could explain the null results.

First, regarding the implementation context, this study was conducted prior to the rapid expansion of RPM technologies to support virtual health care delivery, partly driven by the COVID-19 pandemic in 2020. In the first phase, the investigators relied on PCPs to review, interpret, and integrate the self-measured BP values into their treatment decisions. It is not surprising that PCPs had a low uptake of RPM. For PCPs, managing RPM data requires added work outside of routine clinical encounters (ie, new workflow) or added burden during clinical encounters, which disrupts their routine workflow. Task shifting, involved in reviewing RPM data through faxed paperwork, clicking on various parts of the EHR, or logging on to a third-party technology platform, is known to add cognitive burden and contribute to clinician burnout. While the study included social norm nudges to prompt PCP medication adjustments, the context matters here as well. Although social norm messaging has been shown to influence behavior, effects may have been minimized in this trial due to the larger context in which clinicians are now overburdened by reminders and messages.

For this reason, we and others have shown that team-based care models that delegate RPM implementation to nonphysician practitioners, such as nurses or clinical pharmacists, is essential to effective clinical integration of RPM.^4^ In the second phase, the investigators used a nurse-led model to implement the RPM program, but it did not improve the success of the intervention. This lack of improvement is difficult to explain without additional data. For example, how well were the RPM workflows integrated into the centralized nurse workflow? What were the incentives at the individual and system level for active management, and how well did these additional RPM workflows align with established care responsibilities? These operational factors need to be carefully measured when studying RPM implementation. The study also lacked any description of the training or support given to the practice staff to facilitate adoption of the intervention. Our own work shows that a research-practice alignment is critical to support buy-in and ensure the intervention is compatible with practice workflows and is implemented with high fidelity. While it was unclear how home BP readings were transmitted to the clinic, fundamentally, patients also require significant support in using RPM devices and syncing readings to their patient portal. These digital requirements can be difficult to navigate and are compounded when digital literacy is low and/or patients live in areas that lack broadband access or in-home high-speed internet connectivity.^3^

This study additionally evaluated the effect of a strategy from behavioral science, facilitated cheerleading, which influences behavior through social accountability. In the RPM plus social partner arm, messages were designed to encourage patients to continue their good work or to try to do better in the following week, depending on their performance. However, the weekly feedback message was sent on behalf of the support partner, not written by them, a major limitation. Strategies from the behavioral sciences that leverage social factors can have powerful effects on behavior; however, the implementation context, and a myriad of decisions regarding their execution, can equally influence their effectiveness in certain settings, use cases, and populations.^5^

While the patient population for this trial is notable, as the foundational behavioral science research that forms its principles was conducted almost entirely with White, US college students, there are many limitations to their approach.^6^ For example, the support partners did not directly observe participant’s behavior, had no visibility into the primary outcome, and did not provide feedback directly to participants. Improving our understanding of the efficacy of behavioral techniques on diverse populations is important for the field. Successful trials are likely to include direct observation of participant behavior, participant commitment to the behavior, and personal communication from the support partner.

Finally, the overall lack of implementation process measures leaves a crucial unanswered question: are the negative findings due to poor implementation or an ineffective intervention? Answering this question and moving the field forward requires tracking and analyzing key implementation metrics for continual improvement, such as the protocol for and consistency in patient BP submissions, patient engagement with care, the number of support partners who opted out, clinician engagement with RPM (access and review of RPM data, response to nudges, adoption or treatment recommendation and frequency of treatment intensification), and what data guided the decision to change to a nurse-led model, among others. This study contributes to our understanding of the complexity of implementing RPM-supported hypertension management guidelines and reminds us of how much of the devil is in the details.
